# Exploring the advantages of intensity-modulated proton therapy: experimental validation of biological effects using two different beam intensity-modulation patterns

**DOI:** 10.1038/s41598-020-60246-5

**Published:** 2020-02-21

**Authors:** Duo Ma, Lawrence Bronk, Matthew Kerr, Mary Sobieski, Mei Chen, Changran Geng, Joycelyn Yiu, Xiaochun Wang, Narayan Sahoo, Wenhua Cao, Xiaodong Zhang, Clifford Stephan, Radhe Mohan, David R. Grosshans, Fada Guan

**Affiliations:** 1 Department of Radiation Physics, The University of Texas MD Anderson Cancer Center, Houston, TX 77030 USA; 2 Departments of Radiation and Experimental Radiation Oncology, The University of Texas MD Anderson Cancer Center, Houston, TX 77030 USA; 3 Center for Translational Cancer Research, Texas A&M Health Science Center, Institute of Biosciences and Technology, Houston, TX 77030 USA; 4 Department of Radiation Oncology, Ruijin Hospital, Shanghai Jiaotong University School of Medicine, Shanghai, 200025 China; 5 Department of Nuclear Science and Engineering, Nanjing University of Aeronautics and Astronautics, Nanjing, 210016 China; 6 Department of BioSciences, Rice University, Houston, TX 77005 USA

**Keywords:** Translational research, Computational biophysics

## Abstract

In current treatment plans of intensity-modulated proton therapy, high-energy beams are usually assigned larger weights than low-energy beams. Using this form of beam delivery strategy cannot effectively use the biological advantages of low-energy and high-linear energy transfer (LET) protons present within the Bragg peak. However, the planning optimizer can be adjusted to alter the intensity of each beamlet, thus maintaining an identical target dose while increasing the weights of low-energy beams to elevate the LET therein. The objective of this study was to experimentally validate the enhanced biological effects using a novel beam delivery strategy with elevated LET. We used Monte Carlo and optimization algorithms to generate two different intensity-modulation patterns, namely to form a downslope and a flat dose field in the target. We spatially mapped the biological effects using high-content automated assays by employing an upgraded biophysical system with improved accuracy and precision of collected data. *In vitro* results in cancer cells show that using two opposed downslope fields results in a more biologically effective dose, which may have the clinical potential to increase the therapeutic index of proton therapy.

## Introduction

Worldwide the number of proton therapy centers has increased dramatically in recent years^1^. The expansion of proton therapy centers can be attributed to multiple factors. The first and foremost advantage of protons is that a Bragg-peak dose profile appears at the end of the range of a proton beam. The use of different beam modulation and shaping techniques makes it possible to deliver a uniform high dose to the tumors while sparing the surrounding normal tissues^2^. The uniform target dose can be achieved using the passive-scattering proton therapy (PSPT) technique or the more advanced intensity-modulated proton therapy (IMPT) technique employing scanned beams. Importantly, some clinical trials have indicated the advantages of proton therapy over traditional photon-based therapy^3–6^. However, there are still many challenging issues in modern proton therapy. One of the them is that the radiobiological characteristics of proton therapy have yet to be completely understood. In current clinical practice, the relative biological effectiveness (RBE) of protons to reference photons, such as x-rays from a medical LINAC, is assumed to be a constant of 1.1, which indicates that to achieve the same therapeutic effect to the tumors the photon dose must be 10% higher than the proton dose^7^. The constant RBE of 1.1 was concluded from previous *in vivo* and *in vitro* experiments mainly using the PSPT technique at the middle of a spread-out Bragg peak (SOBP)^8,9^. However, modern particle therapy is increasingly adopting IMPT and this delivery technique will be predominant in the near future. Conclusions derived from PSPT measurements may not be easily translatable to IMPT. With the prevalence of IMPT and the advancement of experimental techniques in radiobiology, the understanding of the proton biological effects has been improved. The use of RBE 1.1 is increasingly questioned. Now, it is well known that proton RBE is a complex function of many physical and biological factors such as dose, cell and tissue types, beam quality, and biological endpoint^9–11^. Nevertheless, the spatially variable RBE has yet been applied clinically in the optimization of treatment plans.

The task group report (TG-256) from the American Association of Physicists in Medicine (AAPM) has summarized the existing problems of proton RBE and provided suggestions for future research directions^7^. This report indicates that there is a very large spread in measured RBE values, due mainly to inconsistent methods used by different teams. This report points to the need for experimental protocol standardization. It also concludes that the continued use of a generic constant RBE of 1.1 may lead to sub-optimal plans with heterogeneous biological effects in the tumor. Further, the interpretation of clinical response data may be misleading. Nevertheless, the report suggests that, for the time being, the use of a constant RBE should continue in clinical practice because the underlying biophysical bases of proton therapy are not well understood. This knowledge gap motivated us to investigate the biological characteristics of protons, especially with scanned proton beams, because such beams are used in IMPT. The novel experimental methods developed by our team and preliminary data will be presented in this report.

The physical quantity linear energy transfer (LET) is frequently used to characterize the beam quality of charged particles. Within the energy range of clinical protons, LET is inversely related to beam energy (neglecting the effect of effective charge change). However, it must be noted that the passage of protons through a medium leads to a spreading of the energy spectrum, resulting in a wider LET distribution, especially at the end of range^12^. To simplify the correlation between physical quantities and biological effects, the concept of dose-averaged LET (LET_d_) has been introduced to obtain the mean value of a LET spectrum using the dose contribution of each LET event as the weighting factor^13^. The definition of LET_d_ indicates that it is not a physical quantity that can be measured directly. LET_d_ is usually calculated using Monte Carlo particle tracking methods or analytical methods^12,14–17^. The results of recent experiments have shown the increasing trend of RBE along the path of mono-energetic proton beams: slowly increasing in the entrance, but sharply increasing in the Bragg peak region^18–21^, which is similar to the spatial variation trend of LET_d_^12^. Therefore, it is believed that LET_d_ may serve as a quantity connecting physical characteristics of beams and the biological effects in the biological samples. LET_d_ has been used in many phenomenological models to calculate proton RBE, such as those developed by Wilkens *et al*.^22^, Carabe *et al*.^23^, Wedenberg *et al*.^24^, and McNamara *et al*.^25^. The use of LET_d_ as a “quantitative” indicator of RBE is controversial due to the large discrepancies between experimental results and predictions from the LET_d_-based RBE models^2,26–29^. In the present study, LET_d_ is only used to “qualitatively” interpret experimental results. Our previous Monte Carlo calculation results show that at the end of range, the maximum LET_d_ of a low-energy incident beam is higher than that of a high-energy beam^30,31^. This indicates that at the end of range, a low-energy incident beam may be more biologically effective than a high-energy beam. This phenomenon provides a straightforward indication that in an IMPT plan, increasing the intensities or weights of low-energy incident proton beams in the target may have the potential to enhance the biological effects therein.

In current IMPT treatment plans, by default, high-energy beams are usually assigned larger weights than low-energy beams^32,33^. Consequently, low-energy beams contribute less to the overall dose in the target volume. Therefore, in its current form, this beam delivery strategy cannot effectively make use of the biological advantages of low-energy (high-LET) protons present within the Bragg peak region. However, the planning optimizer can be adjusted to alter the intensity of each beamlet, thus maintaining the same target dose delivered from multiple fields while increasing the weights of low-energy beams thus enhancing the LET_d_ within the target, so that the biological advantages of protons may be exploited. Bassler *et al*. have investigated the LET painting technique using carbon and oxygen ions to place more high-LET particles in target tumors in the plan optimizations^34,35^. They concluded that using the LET painting can increase the tumor control probability in hypoxic tumors. Fager *et al*. adopted a “split target planning” method to enhance the LET_d_ distributions in the target tumor by splitting the target into different sub-targets. The rationale is to deliver a uniform RBE-weighted dose (=RBE × dose), viz., with expected equivalent clinical effectiveness, to the target with elevated LET_d_ but reduced physical dose in the target. However, it is difficult to evaluate its clinical outcome because the accuracy of the applied RBE model has not been experimentally validated^23^. Tseung *et al*. used a simplified method to perform the RBE-weighted dose optimization for protons. They assumed the RBE is linearly proportional to LET_d_ plus a constant coefficient, disregarding all of other biophysical factors. In the plans generated using their method, an elevated LET_d_ distribution in the target was found compared with to regular IMPT plans. Nevertheless, the simplified relationship between RBE and LET_d_ has made it difficult to evaluate the clinical potential of their plans. Cao *et al*. have developed a method to simultaneously optimize the dose and LET_d_ distributions of IMPT plans. Compared with the conventional IMPT plans, the plans generated using this LET_d_ optimization strategy have elevated LET_d_ in the target tumors and reduced LET_d_ in the critical structures while maintaining the uniform physical dose distribution in the target tumors^36^.

Although many efforts have been made in treatment planning to elevate LET_d_ in the target tumor, the enhanced biological effects have seldom been experimentally validated. In an experimental setup to irradiate cells in the target region, the uniform target dose can be delivered using different IMPT intensity-modulation pattens with multi-field optimizations as long as the optimizer converges on a solution of the beam weights. The simplest and most straightforward method is using two opposed fields for dose patching to deliver the uniform target dose. Two simple solutions of beam weights are presented in this study. One is from two opposed flat dose profiles, and the other is from two opposed downslope dose profiles. The Monte Carlo simulation and dose optimization results in our previous study have shown that using downslope fields elevates the LET_d_ values within the central region of the target^31^. The purpose of the present study was to experimentally validate the hypothesis that using the beam delivery strategy with two opposed downslope dose fields enhances the biological effects in the target region compared to the traditional method using two opposed flat SOBPs. A novel 3D-printed and water-filled irradiation device was designed and applied in the cell irradiation experiments to reduce the systematic uncertainty of experimental data associated with the irradiation device. The combined use of this multi-step irradiation device and the 96-well plates forms the foundation of our high-throughput cell irradiation experiments to spatially map the biological effects along the beam path.

To quantify the biological effects, both clonogenic survival and the DNA damage repair responses were investigated. The non-small cell lung cancer (NSCLC) H460 cell line was selected in the irradiation experiments because of its exceptional clonogenicity and its doubling time, which we found to be approximately 19 hours, in agreement with published values^37,38^. DNA double strand breaks (DSB) were evaluated by quantifying the established DSB repair marker 53BP1 using immunofluorescent (IF) staining. Only the persistent foci (24 hours post irradiation) results are reported in this study. The DNA damage repair kinetics at different time points post irradiation as a function of LET_d_ from a mono-energetic beam can be found in our previous work^39^.

In addition to the experimental measurements, the RBE model developed by McNamara *et al*. has been used to predict the proton RBE for the present experimental setup and to generate the predicted cell surviving fractions which are then compared with the experimental data in this study. Additionally, we will also discuss the clinical potential of using the two downslope fields by comparing the treatment plans using the two different scan patterns for a prostate cancer patient case using IMPT optimization algorithms developed by Cao *et al*.^36^.

## Results

The basic physics principle of the experimental setup is to use two opposed proton fields to form a uniform dose region longitudinally to cover the target. In addition to the requirement of the longitudinal dose uniformity in the target, the lateral dose profiles are also required to be uniform and large enough to cover the target volume. In the experimental setup for cell irradiations, two 96-well plates are used in each irradiation. The dimension of one plate is 12.7 cm × 8.5 cm. The scan patterns have been designed to form a uniform dose area of 17 cm × 17 cm laterally, which is large enough to cover two plates in the experiment. The lateral dose profiles from measurements can be found in the Supplementary Materials.

### Longitudinal dose and LET_d_ distributions from a single IMPT field

**Figure 1:**
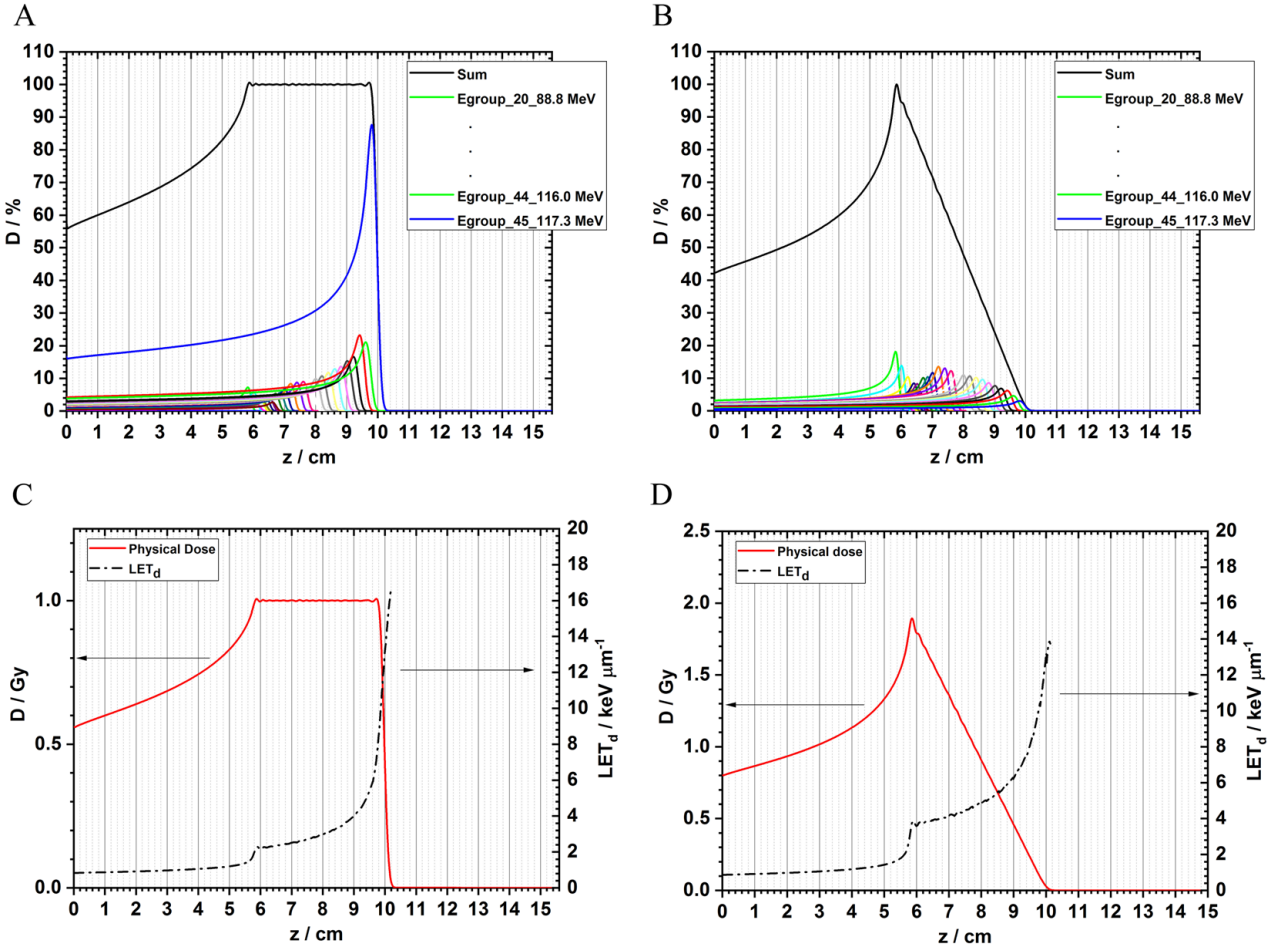
Dose and LET_d_ distributions from a single flat SOBP and a downslope SOBP. (**A**) The dose of a flat SOBP and its beam constituents. The higher energy beams contribute more to the total dose. (**B**) In a downslope target dose profile, the intensities of the lower energy beams are higher than in panel (A). (**C**) The LET_d_ distribution from the flat SOBP. (**D**) The LET_d_ distribution from the downslope field.

In total, 94 energies of scanned proton beams are available at The University of Texas MD Anderson Cancer Center Proton Therapy Center. The dose and LET_d_ distributions in water for each beamlet have been pre-calculated using Monte Carlo simulations^30,31^. An in-house dose optimization algorithm has been developed using the Python programming language to generate the desired dose distribution profiles^30,31^. Figure 1A shows a flat SOBP (the target region of 5.8 to 9.8 cm) and the dose contributions from its constituent beams. The beam with the highest energy contributes most to the total dose. Figure 1B shows a downslope SOBP (the target region of 5.8 to 9.8 cm) and the dose contributions from its constituent beams. In both dose profiles, 26 energies from 88.8 MeV to 117.3 MeV are used. In the downslope SOBP it is clear that low-energy proton beams contribute more to the total dose than within a flat SOBP. The LET_d_ distributions from these two beam delivery strategies are compared in Fig. 1C,D. The LET_d_ increases from 1.98 keV/μm (at 5.8 cm) to 8.57 keV/μm (at 9.8 cm) within the flat SOBP. In contrast, within the downslope SOBP, the LET_d_ has been elevated, increasing from 3.15 keV/μm (at 5.8 cm) to 10.32 keV/μm (at 9.8 cm).

### Longitudinal dose and LET_d_ distributions from two opposed fields for the cell irradiation experiments

**Figure 2:**
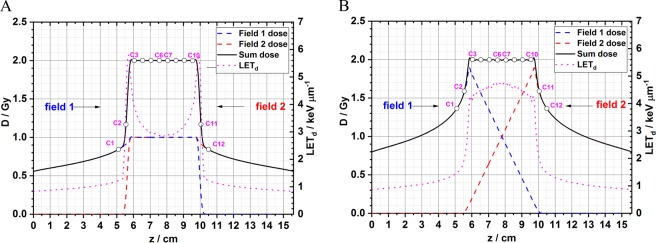
The dose and LET_d_ distributions in water from two opposed fields. (**A**) The total dose is superimposed by two opposed flat dose profiles. (**B**) The total dose is superimposed by two opposed downslope dose profiles. C1, C2, etc. stand for the column #1, #2, etc., of a 96-well plate.

In the cell irradiation experiments, only the role of LET_d_ in the biological response is evaluated, therefore, all other parameters, including the dose, were fixed. A straightforward experimental method is to place the cells within the uniform dose region formed by two opposed dose fields. The uniform dose region (5.8 to 9.8 cm) formed by the two opposed flat dose SOBPs and the two opposed downslope dose SOBPs are shown in Fig. 2A,B. 2 Gy is used as the target dose in the plots for demonstrative purposes but other doses can be delivered by changing the monitor units in the scan patterns. LET_d_ distributions are also included, marked by the magenta dashed lines. The curves show that using the two opposed downslope dose SOBPs can provide elevated LET_d_ distributions within the target region. The purpose of this study is to experimentally quantify the difference in the biological effects using these two different beam delivery strategies and to evaluate the clinical potential using the scan pattern with the elevated LET_d_ distribution. The cell irradiation experiments in the present study were performed using the high-throughput method developed by our team with a custom multi-step irradiation device and 96-well plates. The details of the experimental design can be found in the section of Materials and Methods. Twelve locations along the sum dose curve are selected corresponding to the twelve columns of a 96-well plate. As indicated in Fig. 2, the middle eight columns are placed within the target region of uniform dose. The outside four columns are only used for reference and the experimental results are not reported because the dose levels are not identical in these two setups. This is a symmetric design about the physical center of the 96-well plate with the middle point at the depth of 7.8 cm in water. For example, column 1 and 12 receive the same dose and LET_d_; column 6 and 7 receive the same dose and LET_d_, etc. Only the biological effects in the middle eight columns are compared because they have the same dose but different LET_d_ values (Table 1**)**.

**Table 1:** The LET_d_ values of the middle eight columns in a 96-well plate within the uniform dose region.

LET_d_ (keV/μm)	Col 3&10	Col 4&9	Col 5&8	Col 6&7
Flat	3.93	3.23	2.98	2.87
Downslope	4.20	4.49	4.65	4.68

### Clonogenic survival from reference photons

**Figure 3:**
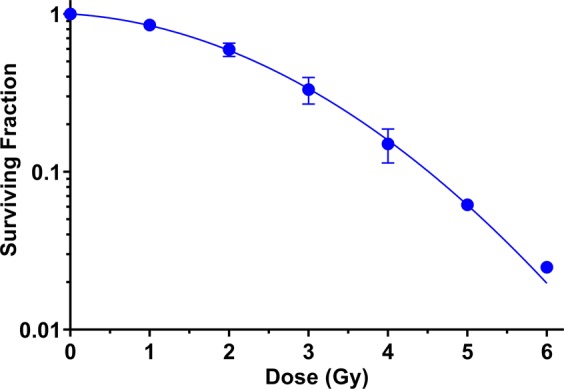
The survival curve of H460 cells from reference photons. At some data points, the error bars are too short to be plotted.

In addition to the proton irradiations, photon irradiations were performed synchronously using the gamma rays from a Cs-137 irradiator or 6 MV x-rays from a medical LINAC. The clonogenic survival curve of H460 cells from experiments (n = 3 repeats, twice using Cs-137 and once using 6 MV) is plotted in Fig. 3. The error bar of the surviving fraction (SF) shows the standard error of mean (SEM). The survival curve was fit to the linear-quadratic (LQ) model expression as in Eq. (1).

1 $$SF=\exp (-\alpha D-\beta {D}^{2}),$$

where α and β are parameters solved from curving fitting and D is dose. The fitting results are α = 0.0723 ± 0.0285 Gy^−1^ and β = 0.0971 ± 0.0083 Gy^−2^. These values can be used in the phenomenological McNamara model for proton RBE calculations. The details of the calculation method can be found in the Supplementary Materials. The experimental SF (mean ± SEM) at 2 Gy and 4 Gy is 0.595 ± 0.033 and 0.150 ± 0.021. These values will be compared with the clonogenic survival results from proton irradiations in the next section.

### Clonogenic survival from protons

**Figure 4:**
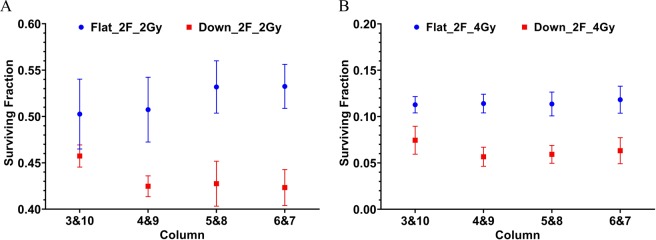
Surviving fractions in the middle eight columns from the two different scan patterns of two opposed flat fields and two opposed downslope fields. (**A**) The results from 2 Gy. (**B**) The results from 4 Gy. The error bar is the SEM from 3 repeated experiments.

Scan patterns and the irradiation device were customized to deliver a uniform dose to a 4-cm wide target (5.8–9.8 cm in water) covering the middle eight columns of a 96-well plate seeded with lung cancer H460 cells. Two 96-well plates were exposed simultaneously in each irradiation. The results of clonogenic survival of H460 cells following irradiation with 2 Gy and 4 Gy are presented here. The results are expressed as the mean ± SEM. The results are from 3 repeated experiments, and in each experiment the surviving fraction is the average value from 32 replicates (8 wells per column × 4 columns in two plates). The surviving fractions at 2 and 4 Gy are shown in Fig. 4A,B, respectively. The results show that the overall surviving fraction using the two opposed downslope fields (red) is lower than using the two opposed flat fields (blue). The results experimentally validate the hypothesis of the present study that using the downslope fields can enhance the biological effects than using the traditional flat fields.

**Figure 5:**
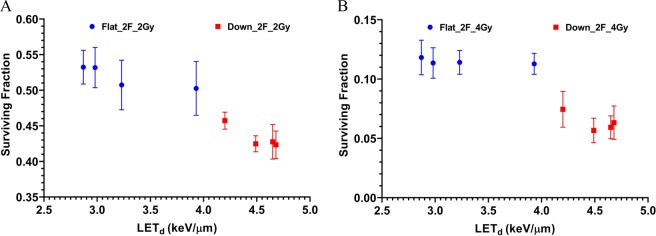
The surviving fraction as a function of LET_d_ from the two different scan patterns of two opposed flat fields and two opposed downslope fields. (**A**) The results from 2 Gy. (**B**) The results from 4 Gy. The error bar is the SEM from 3 repeated experiments.

**Figure 6:**
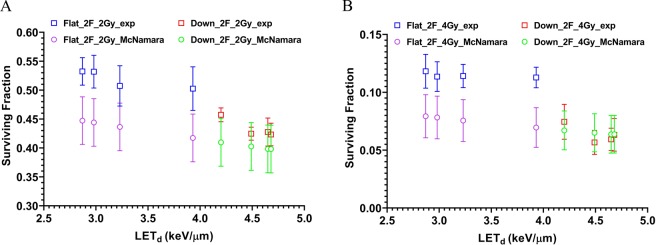
The surviving fraction as a function of LET_d_ from experiments and McNamara model predictions. (**A**) The results from 2 Gy. (**B**) The results from 4 Gy.

The surviving fraction was then re-plotted as a function of LET_d_ for all data from both setups, as shown in Fig. 5A,B. The general trend is that the surviving fraction decreases with the increase of LET_d_. In addition, using the McNamara RBE model with the input parameters of proton dose, LET_d_, the α and β of the survival curve of reference photons (Fig. 3), the cell surviving fractions with proton irradiations were calculated. The experimental data and model predicted SF as a function of LET_d_ are compared in Fig. 6. The McNamara model was only used to predict proton RBE. The cell survival of protons was derived from the RBE-weighted dose and the LQ survival curve of reference photons. For the McNamara model results, the uncertainty is derived from error propagation including all the errors of the variables in the McNamara formula and the standard errors of α and β from the survival curve fitting of the reference photons. The details of the model calculations can be found in the Materials and Methods section. The survival data of Fig. 6 are listed in Table S1 in the Supplementary Materials.

### Persistent 53BP1 foci from protons

**Figure 7:**
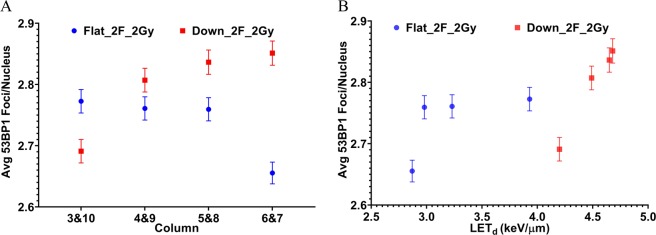
The persistent foci data (expressed as average number of foci per nucleus) with 2 Gy at 24 hours after proton irradiation. (**A**) The data in the middle eight columns from the two different scan patterns of two opposed flat fields and two opposed downslope fields. (**B**) The foci data are replotted as a function of LET_d_.

To demonstrate the applicability of the developed system for use with assays other than clonogenic survival, we next compared DNA double-strand break repair (measured as 53BP1 foci formation) using these two different scan patterns. The DNA damage response is a complex process involving numerous proteins and effectors. DSBs are the most difficult DNA lesion to repair and are thus considered the most lethal form of DNA damage. Markers of DNA damage assessment include the repair proteins which accumulate at the site of a DSB. When imaged these proteins appear as small foci when the DNA damage is localized within a small region of the nucleus subsequently recruiting repair proteins. DNA damage repair kinetics following irradiation can be measured by fixing cells at different time points post-exposure, labeling the repair proteins, and scoring the number of foci present. Two well established ubiquitous DSB markers are γH2AX and p53 binding protein 1 (53BP1)^40,41^. DSB repair kinetics have been investigated in our previous study^39^. Here, only the persistent foci data (expressed as average number of foci per nucleus) with 2 Gy at 24 hours after proton irradiation are presented, as shown in Fig. 7A. Except in column 3&10, in all other columns, downslope fields resulted in increased foci present at 24 hours post-irradiation. The foci present is also plotted as a function of LET_d_ in Fig. 7B. In general, the foci data show the increasing trend with LET_d_ except the point with LET_d_ = 4.20 keV/μm. The number of 53BP1 foci per nucleus (at 2 Gy at 24 hours after proton irradiation) as a function of LET_d_ are listed in Table S2 in the Supplementary Materials.

## Discussion

In many charged particle radiobiology experiments, plastic phantoms are used as the range shifters to obtain biological effect data at different spatial locations along the beam path. The efficiency of this traditional method is relatively low and the accuracy of the experimental data may have relatively large uncertainties caused by the conversion of plastic phantom to water equivalent thickness. In the present study, we have refined a high-throughput biophysical system originally developed by our team to investigate the biological effects of proton therapy using scanned proton beams^18^. As with previous versions, this upgraded system can be used to obtain proton biological effect data for different assays such as quantifying the cell clonogenic survival or investigating DNA damage responses. Simultaneously irradiating two 96-well plates increased the number of available replicates resulting in increased experimental data precision. Furthermore, using the high-throughput method, we obtain large amounts of the experimental data in one irradiation with different physical parameters, thus improving the efficiency of the experiments. Aside from improving the efficiency in obtaining biological effect data, this newly designed irradiation system improves the accuracy and precision of the experimental data by directly using water as the primary beam attenuation material as opposed to Lucite or other plastics which serves to reduce systematic uncertainty caused in the conversion of solid material thickness into water equivalent thickness.

In a previous study, Elasser *et al*. developed an acrylic phantom to validate accuracy of the local effect model (LEM) for RBE predictions of protons, carbon ions, and helium ions^42^. By housing multiple plastic inserts plated with cells in the phantom, biological effect data at different locations along the beam path were obtained within a single exposure. Using this device greatly improved the efficiency in obtaining data compared with the traditional exposure method; however, the overall system consisted of mostly plastic, which could cause systematic uncertainties. An additional limitation of the plastic insert system was the low spatial resolution of in that it could only sample the biological effects of fixed large spacings, i.e., 5 mm. In contrast, the irradiation systems designed by our team can spatially map the biological effects with sub millimeter resolution, which is highly useful in the Bragg peak region and distal edge where the dose gradient is high^18^.

In addition to the advantages mentioned above, the irradiation system we have developed is versatile in that it can be used for multiple purposes such as mapping the biological effects along a mono-energetic beam path or poly-energetic beams as in this study. By designing new jigs with different step thicknesses and different spatial resolutions, the biological effects from any beam energy can be investigated. This irradiation system can also be used to validate the accuracy of RBE models providing the scan patterns from uniform RBE-weighted dose plans (inhomogeneous physical dose distribution) for any RBE prediction models^31,43^. Moreover, this irradiation system can easily be translated to investigate the biological effects of other ions such as helium and carbon ions.

One of the long-term goals of the whole proton therapy research community is to apply accurate biological dose optimized IMPT plans in clinic. However, the large discrepancy between RBE model predications and experimental data has impeded such progress^29^. The experimental data from the present study have shown the enhanced biological effects using the two opposed downslope dose fields with increased LET_d_ distribution in the target. However, using LET_d_ as a quantitative input parameter of a phenomenological RBE model, such as the McNamara model, cannot accurately predict the experimental results as shown in the present study and previous studies^18,25^. The reason for this discrepancy is not completely understood, but it may be caused by the “averaged” nature of the physical quantity LET_d_ which completely neglects the spectral distribution of energy deposition events^28^. Understanding the underlying reasons why there are large discrepancies between experimental data and model predictions is an ongoing work within the field.

Applying the stochastic quantity lineal energy in microdosimetry and considering the spectral distribution of lineal energy may better correlate biological outcome with physical interactions between ionizing radiations and biological systems^28^. The microdosimetric spectra can be measured using a tissue-equivalent proportional counter (TEPC)^44–46^ and from Monte Carlo simulations^47–50^. Comparing the predictions using microdosimetry-based RBE models such as MKM^51–53^ and RMF^54–56^ with experimental data is an ideal future application of our high-throughput system.

Our previous studies have shown that for NSCLC H460 and H1437 cell lines, the RBE vs LET_d_ trends are similar. In our future studies, we intend to expand the number of biological models to further validate the hypothesis of the present study. Building a biological database for different cell lines can be used to better understand the underlying mechanisms of proton biological effects and to improve the accuracy of existing RBE models or develop new ones.

In addition to the clonogenic results presented, mechanistic DNA damage response studies will be further investigated. Markers downstream of the initial damage response may be used to differentiate between the DNA damage repair pathways to validate and further elucidate the beam quality-dependence for the formation of persistent foci. Markers of particular interest would be DNA-PKcs for non-homologous end joining (NHEJ) and Rad51 for homologous recombination (HR)^57^.

Moreover, small animal irradiation experiments, i.e., using syngeneic mouse tumor models, will be performed to investigate the difference of the *in vivo* response using the two different scan patterns but with a much narrower target region as proposed in our previous study^31^. Additionally, the experimental approach developed in this study can be also extended to other applications such as investigating the immunologic characteristics of charged particle therapy to determine if high-LET particles can increase immunogenic cell death and the adaptive immune response.

In summary, the results from the current *in vitro* study have experimentally validated that using two opposed downslope dose fields results in more biologically effective radiation dose in the target region than using the traditional two flat dose fields. Our results also show large discrepancies between experimental data and model predictions in clonogenic cell survival. Comparing the experimental results with other RBE models and understanding the reasons for the limitations of RBE models will be the focus of our future studies. Our experimental results further consolidate the statement that the beam delivery strategy with elevated LET_d_ distributions has the potential for improving the therapeutic index in clinical practice, e.g., treatment for prostate cancer patients where parallel opposed fields are typically used. Nevertheless, the dose patterns in Fig. 2A,B show that using the two opposed downslope fields may deliver slightly higher doses to the regions outside of the target, which are usually normal tissues and organs. Therefore, plans generated with two opposed downslope fields should be evaluated to determine if the dose to normal tissues is still below the threshold value in the guideline for IMPT plans. Additional steps, such as the application of dose constraints or additional fields during treatment planning might also mitigate this increase in dose. The evaluation of clinical potential of treatment plans with an elevated LET_d_ distribution for a prostate patient case can be found in the Supplementary Materials.

## Methods

### Design of the beam delivery scan patterns

The dose and dose-averaged linear energy transfer (LET_d_) distributions of all of the 94 scanned proton beams at The University of Texas MD Anderson Cancer Center Proton Therapy Center have been pre-calculated using the Monte Carlo simulation toolkit Geant4^30,31^. It should be noted that the LET_d_ results in our simulations do not take into account the contributions of nuclear fragments and recoil nuclei from the hadronic physics processes. The reasons are twofold. First, according to the definition, the physics quantity of LET is defined for a specified type of charged particle and therefore it is inappropriate to mix the LET values from different types of charged particles^12,58^. Second, LET is strictly limited to the energy loss of charged particles from the “electronic” interactions which include ionization and excitation processes only^12,58^. An in-house C++ code was used to generate the large-field laterally uniform dose for each energy layer. The uniform dose area is 17 cm × 17 cm, large enough to cover two 96-well plates. A dose optimization algorithm for IMPT has been developed by our team using the Python programming language to generate the desired dose distribution for radiobiological studies^30,31^. In the present study, one 4-cm wide flat SOBP and one downslope SOBP were generated ranging from 5.8 to 9.8 cm in water, as shown in Fig. 1. Two opposed flat dose profiles or two downslope dose profiles were combined to form a uniform dose region centered at 7.8 cm. Next, an in-house C program was used to generate the beam delivery scan patterns including the information of beam energies, spot locations, and monitor unit per spot, etc., which can be read in later by the proton beam delivery system.

### Design of a novel proton irradiation system and the experimental setup

**Figure 8:**
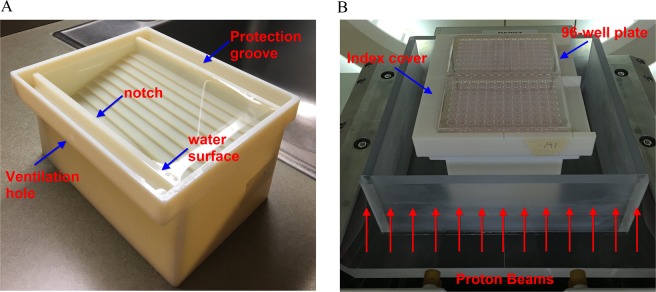
The 3D-printed irradiation device and the experimental setup of cell irradiations. (**A**) The 3D-printed multi-step irradiation device filled with water. (**B**) The experimental setup with two 96-well plates (for clonogenics as the demonstration) placed on the top of the irradiation device. The plates can be irradiated with the large field of scanned protons from below. The arrows and texts in the images were edited using Snagit Editor.

In our previous studies, Lucite (also known as Acrylic or PMMA) blocks were used as the template to fabricate the stepwise irradiation devices (jigs) to spatially map the biological effects along the beam path using the high-throughput method developed by our team^18,59^. There are twelve steps with different thicknesses in the jig matching the twelve columns in a 96-well plate. In each column, the cells in the eight wells receive the same dose-LET_d_ combination. This experimental design enables the collection of twelve data points along the beam path in each irradiation. The combined use of a multi-step jig and 96-well plates forms the foundation of our high-throughput irradiation strategy for cell experiments using particle beams. In this study, a 3D-printed stepwise “water” container was designed as the irradiation device, shown in Fig. 8A. Using water as the main attenuation medium minimizes the uncertainty introduced by the material conversion from Lucite to water equivalent thickness (WET) as used in our previous experiments and by many other teams, thereby improving the accuracy of experimental results. Prior to the cell irradiations, the jig is first filled with water until the water surface reaches the notch as indicated in Fig. 8A. Air bubbles produced during the filling process are manually cleared. The water surface is convex relative to the notch due to surface tension when the jig is full of water. The plate holder cover is placed on top of jig to force the extra water into the protection grooves to make the water surface flush with the bottom of the index cover. The ventilation holes allow air to be expelled when the index cover is pushed down. The use of the index cover reduces the random uncertainty when water is used as the stopping medium. The experimental setup is shown in Fig. 8B, where two 96-well plates are placed on the top of the index cover. The material of the device is digital ABS (measured ρ = 1.164 g/cm^3^). The device was printed out using a Stratasys J750 printer with a 14 μm resolution. The WET of each step, including the jig holder, the bottom of each step (digital ABS), water, the index cover (digital ABS), and the bottom of a 96-well plate, is designed to be equal to the depth in water as demonstrated in Fig. 2. Two index covers are customized to compensate the difference of the WET of the different types of 96-well plates used for clonogenics (polystyrene bottom) or immunofluorescence (glass bottom).

The purpose of using this irradiation device is to deliver a uniform dose to the middle eight columns of a 96-well plate from “two” opposed fields; therefore, the columns are distributed symmetrically. In this design, the symmetric axis is the center of the SOBP, which is 78.0 mm. The WET values of the twelve locations are listed in Table 2. Column 1 and column 12 are symmetric; the column 2 and column 11 are symmetric, and so on. The total dose in each column of the 96-well plate is actually from two irradiations using the same scan pattern. Taking column 3 as the example, in the first irradiation, the column receives the dose at the WET of 60.5 mm while its symmetric location, column 10, is exposed at a WET depth of 95.5 mm. The plate is then rotated by 180 degrees so that in the second irradiation using the same scan pattern, column 3 is at a WET depth of 95.5 mm and column 10 receives the dose at the WET of 60.5 mm. Consequently, the final dose for each column is the sum of dose from two symmetric locations centered at 78.0 mm in the dose curve of a single field. However, the uncertainties (systematic and random) of the WET of each step could bring uncertainties to the dose and LET_d_ in each column. The details of the uncertainties are described in the section of sensitivity analysis.

**Table 2:** The water equivalent thickness of each step for the experimental setup.

Column	1	2	3	4	5	6	7	8	9	10	11	12
WET (mm)	51	55.5	60.5	65.5	70.5	75.5	80.5	85.5	90.5	95.5	100.5	105

Because of the symmetric design of the experimental device, two plates per irradiation generate 32 replicates (8 wells per column × 4 columns in two plates) for each condition (same dose and same LET_d_) as listed in Table 1, further improving the efficiency of experiments and the precision of experimental data.

### Validations of the proton dose distributions

The depth dose distributions from a single field of the two scan patterns as shown in Fig. 1 were derived from the Monte Carlo calculations and IMPT optimizations. Quality assurance (QA) measurements must be performed before using these scan patterns for cell irradiations. The QA results using the Zebra multi-layer ion chamber (IBA Dosimetry, Schwarzenbruck, Germany) for depth dose (relative) distributions are shown in Fig. S1 in the Supplementary Materials. For both scan patterns, the measured dose at any depths within the SOBP was within the +/−2% lines (black dash) of the Monte Carlo calculated dose.

In addition, the lateral 2D dose maps were measured using the MatriXX ion chamber array (IBA Dosimetry, Schwarzenbruck, Germany) with solid water blocks as buildup to test the dose uniformity at different depths. Three different locations within the SOBP were selected, but only the measurements at the middle point of the SOBP are presented. The 1D dose profile along the central x and y axes extracted from the 2D dose maps are shown in Fig. S2 in the Supplementary Materials. For both scan patterns, the 1D dose profile along either central axis is within 98% and 102% dose lines, providing a uniform dose field of 17 cm × 17 cm, large enough to cover two 96-well plates.

For the cell irradiation setup, Gafchromic EBT3 films were placed on the top of an empty 96-well plate (glass bottom plate for immunofluorescence) to measure the optical density profiles, which can be used to represent the dose profiles of the scan patterns. In the measurements, the single-field flat and downslope scan patterns were used respectively to deliver 1 Gy at the middle point of the SOBP, as illustrated in Fig. 1. The optical density profiles can be found in Fig. S3 in the Supplementary Materials, which are similar to the dose curves shown in Fig. 1. The purpose of film measurements in the present work was to test if the middle eight columns are within the flat or downslope SOBP, rather than measuring the absolute dose in the cell layers attached to the bottom of each well. The details for converting the optical density to the absolute dose for protons can be found in the references^60,61^.

The absolute dose above the jig without the 96-well plates was validated using the Advanced Markus chamber (model 34045, PTW-Freiburg, Freiburg, Germany) for select columns. The ion chamber was placed on top of the clonogenic-specific index cover to a specified column during the measurement. The measured dose and MC calculated dose (considering the WET of the chamber window = 1.06 mm) for three sampled columns are compared in Table S3 in the Supplementary Materials. The difference is within ±3% for the selected columns.

### Hitachi proton therapy system

Proton irradiations were delivered using the spot-scanning nozzle with the Hitachi ProBeat delivery system (Hitachi, Ltd., Tokyo, Japan) at The University of Texas MD Anderson Cancer Center Proton Therapy Center^62^. This proton beam delivery system can provide 94 discrete energies ranging from 72.5 MeV to 221.8 MeV^63,64^. It uses a step-and-shoot scanning technique in which the beamlet aims at a specified location and delivers the specified number of monitor units and then moves to the next position. A custom scan pattern containing the information of all of the spot locations and beam energies was uploaded to the system prior to the cell irradiations. In the present experiments, the biological samples were scanned by beamlets with multiple energies. The scanning order of the beam energy layers is from high to low. Only when all of the locations for one specified energy layer were scanned completely, the system could start the scanning for the next energy layer.

### Photon irradiations

The reference photon irradiations were carried out synchronously using a Cs-137 irradiator or a medical LINAC. The Cs-137 irradiator (SN 1138, JL Shepherd & Associates, San Fernando, CA) has a line source, which is moved under the irradiation chamber when the unit is turned on. In-air dose rates were determined by applying the AAPM TG-61 protocol to the ion chamber measurements. The measured values agree with the historic reference value within the acceptable window (±5%).

The 6-MV x-ray beams (Varian 21EX LINAC, Varian Medical Systems, Palo Alto, CA) were also used for cell irradiations. Cells were positioned at the 10 cm water equivalent depth (solid water blocks) in the center of the irradiation field (25 cm × 25 cm field size). The absolute doses from the LINAC were calibrated according to AAPM TG-51 report with the accuracy of 1%. Dose uncertainties arising from cell plate positioning or beam fluctuations were estimated to be within ±2%.

### Sensitivity analysis of experimental setup uncertainties

Given the setup and the uniformity of the irradiation field, the accuracy of the dose delivered to the biological samples is insensitive to small changes in positioning relative to the beam. However, the uncertainties brought by the irradiation system and the 96-well plates influence the accuracy of the dose and LET_d_ values in the target cells. The jig holder, the jig bottom, and the index cover are fixed components once fabricated, so the fabrication accuracy may only bring systematic uncertainties to the experimental data. However, the high accuracy (14 µm) of the 3D printer ensures that the device uncertainty is sufficiently small to be negligible. The jig was filled with water for each experiment and the real thickness may vary between experiments. Although the index cover was used to minimize the variation of water level there may still be the random uncertainty. Manufacturing variability in the thickness of the well bottom for the 96-well plates and the composition also contribute to random uncertainty. Considering all the sources of uncertainty, the maximum change of all of the components in the beam path was assumed to be 1 mm WET. Then ±1 mm was used as the largest shift for the sensitivity analysis of the experimental setup. The estimated uncertainties for dose and LET_d_, based on the MC calculations, to the middle eight columns of the 96-well plate are given in Tables S4 and S5 in the Supplementary Materials. When using two opposed flat fields, the dose variations due to the attenuation components are negligible, but the LET_d_ variations are large with the maximum change of 8.45% in column 3&10 when the WET change is 1 mm. When using two opposed downslope fields, the dose variations due to the attenuation components are within ±5%. However, the LET_d_ variations are not sensitive to the WET change, and they are all within ±3%. The sensitivity analysis results can be explained from the dose and LET_d_ distributions in Fig. 2, which shows that the sensitivity of dose and LET_d_ strongly depend on the gradients of the dose and LET_d_ curves.

### Biological sample preparation, irradiation, and processing

The NSCLC H460 cell line was obtained from American Type Culture Collection (ATCC). H460 cells were cultured in RPMI 1640 medium with 10% fetal bovine serum (FBS) and 1% penicillin-streptomycin in a humidified incubator at 37 °C and 5% CO_2_. Cells were counted using an automated Vi-Cell XR system. For the high-throughput clonogenic assays, cells were plated using a multichannel pipette with 100 cells per well in a total volume of 100 μL. Following the cell plating, 96-well plates required a 1.5-hour incubation at room temperature (RT) for sufficient cell attachment to prevent movement when they are returned to culture at 37 °C^65^. Cells were allowed to attach and stabilize in culture for 8–10 hours before irradiation. Two plates were brought into the treatment room for each proton irradiation with the multi-step water jig and clonogenics-specific index cover and immediately returned to the incubator after exposure. For reference photons, only one plate was irradiated per dose level. Control plates were processed identically to the irradiated plates but without irradiation. After colonies formed (at 5.5 days for the H460 cells), cells were fixed and stained with 0.5% crystal violet in 100% ethanol. A high-content automated laser confocal system (IN Cell Analyzer 6000) was used to identify viable colonies. Only colonies containing 50 or more cells were used for data analysis. The surviving fraction for each well was analyzed by normalizing the number of counted colonies by the plating efficiency of the control plates.

53BP1 foci formation after proton irradiation was examined by plating cells into glass-bottom 96-well plates with 12,000 cells in 100 μL seeded per well, irradiating the plates with the multi-step water jig and the foci-specific index cover, followed by standard culture. For the persistent foci analysis, 24 hours after irradiation, cells were fixed with 4% paraformaldehyde in phosphate buffered saline (PBS). The fixative was removed and cells were washed in PBS 3 times before permeabilizing and blocking with a 5% goat serum/0.3% Triton X-100/PBS solution overnight (14–16 hours). For primary labeling, the cells were incubated at RT for 2 hours at RT. 53BP1 primary antibody used was rabbit polyclonal (ab21083, Abcam). The concentration of the primary antibody used was 0.8 μg/ml. The antibody dilution buffer was 5% goat serum/0.3% Triton X-100/PBS. Following primary antibody incubation, cells were washed five times for 10 minutes at RT with 0.1% Triton X-100/PBS. Cells were then incubated with goat anti-rabbit highly cross-adsorbed secondary antibody conjugated to AlexaFluor594 dye in 5% goat serum/0.3% Triton X-100/PBS at a concentration of 0.5 μg/ml (1:4000, Life Technologies) for 1 hour at RT covered in aluminum foil. Following secondary antibody incubation, the cells were washed with 0.1% Triton X-100/PBS five times for 10 minutes at RT. Nuclei were labeled by incubation with 1 μg/ml of 4′,6-diamidino-2-phenylindole (DAPI) in PBS for 10 minutes RT. The plates were then washed 3 times with PBS for 5 minutes at RT prior to addition of mounting media, the final protocol step. Liquid removal within the IF procedure was achieved using the “swish and flick” method. Solutions within the IF protocol were dispensed using a 12-well multichannel pipette as quickly as possible to ensure samples did not dry.

Plates were imaged on a Cytation5 Cell Imaging Multi-Mode Reader utilizing automated microscopy with a 20× in air objective. The plate geometry was registered within the platform’s Gen5 software. The imaging protocol was set to collect images at 25 locations (5 × 5 grid with 700 μm spacing between each field of view) within each well resulting in 2400 images per channel per plate. At each location a default autofocus scan was performed on the blue (DAPI) channel to determine the scan height. Two images were then sequentially captured corresponding to the fluorescently labeled components in the blue (nuclei) and red (foci) channels. The protocol run time was approximately 4 hours per plate. The images were then exported for analysis.

The general procedure to determine the average number of foci per nucleus was to identify DAPI-labeled nuclei and DSB-repair protein foci as image masks and quantify the number of nuclei-associated foci using CellProfiler 2.2.0 (Broad Institute). The modules and the settings used in the CellProfiler pipeline for the foci analysis can be found in the reference^39^. The settings were manually benchmarked by eye on a subset of the captured images.

### Statistical analyses

Statistical analyses were performed using GraphPad Prism 8.0. SF data from both protons and photons are shown as mean ± standard error of the mean. SF data from reference photons vs. dose were fit using a weighted (1/Y)-nonlinear regression to the linear-quadratic model. The 53BP1 foci data are shown as mean ± standard error of the mean. The standard error of the SF using the LQ model and the McNamara RBE model was calculated by propagating the standard error of the α and β fits in the SF curve of the photons, and the error of the McNamara model-based RBE. The details about calculating the error of the McNamara predicted RBE can be found in our previous publication^31^.

## Supplementary information

Supplementary informations.
