# Author Correction: Exploring the advantages of intensity-modulated proton therapy: experimental validation of biological effects using two different beam intensity-modulation patterns

**DOI:** 10.1038/s41598-020-75965-y

**Published:** 2020-10-30

**Authors:** Duo Ma, Lawrence Bronk, Matthew Kerr, Mary Sobieski, Mei Chen, Changran Geng, Joycelyn Yiu, Xiaochun Wang, Narayan Sahoo, Wenhua Cao, Xiaodong Zhang, Clifford Stephan, Radhe Mohan, David R. Grosshans, Fada Guan

**Affiliations:** 1 Department of Radiation Physics, The University of Texas MD Anderson Cancer Center, Houston, TX 77030 USA; 2 Departments of Radiation and Experimental Radiation Oncology, The University of Texas MD Anderson Cancer Center, Houston, TX 77030 USA; 3 Center for Translational Cancer Research, Texas A&M Health Science Center, Institute of Biosciences and Technology, Houston, TX 77030 USA; 4 Department of Radiation Oncology, Ruijin Hospital, Shanghai Jiaotong University School of Medicine, Shanghai, 200025 China; 5 Department of Nuclear Science and Engineering, Nanjing University of Aeronautics and Astronautics, Nanjing, 210016 China; 6 Department of BioSciences, Rice University, Houston, TX 77005 USA

Correction to: *Scientific Reports* 10.1038/s41598-020-60246-5, published online 21 February 2020

The Acknowledgements section in this Article is incomplete.

“We thank Dr. Ronald Zhu and Dr. Falk Poenisch for their support and guidance in the proton dosimetry. We thank Dr. Sang Hyun Cho for the support in the dosimetry of the Cs-137 irradiator. We thank Dr. David Volk and Mr. Paul Wisdom for fabricating the high-throughput irradiation device and its supporting and protection system. We thank Dr. Aimee McNamara for sharing her knowledge in RBE modelling. We thank Ms. Christine Wogan for the editorial assistance. This work was supported by the National Cancer Institute [Grant No. U19 CA021239] and by the Radiation Oncology Strategic Initiative Boot Walk Award from The University of Texas MD Anderson Cancer Center.”

should read:

“We thank Dr. Ronald Zhu and Dr. Falk Poenisch for their support and guidance in the proton dosimetry. We thank Dr. Sang Hyun Cho for the support in the dosimetry of the Cs-137 irradiator. We thank Dr. David Volk and Mr. Paul Wisdom for fabricating the high-throughput irradiation device and its supporting and protection system. We thank Dr. Aimee McNamara for sharing her knowledge in RBE modelling. We thank Ms. Christine Wogan for the editorial assistance. This work was supported by the National Cancer Institute [Grant No. U19 CA021239], the Cancer Prevention and Research Institute of Texas (CPRIT) [RP150578], and by the Radiation Oncology Strategic Initiatives Boot Walk Award from The University of Texas MD Anderson Cancer Center. JY was supported by the CPRIT Research Training Program [RP170067].”

