# Supplementary material for: Exploring the advantages of intensity-modulated proton therapy: experimental validation of biological effects using two different beam intensity-modulation patterns
Source: Sci Rep. 2020 Feb 21;10:3199. doi: 10.1038/s41598-020-60246-5 (PMC7035246; doi:10.1038/s41598-020-60246-5)
Supplement: Supplementary file 1 — Supplementary informations. [file 41598_2020_60246_MOESM1_ESM.pdf]

Supplementary Materials

Exploring the advantages of intensity-modulated proton therapy: experimental validation of biological effects using two different beam intensity-modulation patterns

Duo Ma[1], Lawrence Bronk[2], Matthew Kerr[1], Mary Sobieski[3], Mei Chen[1,4], Changran Geng[5], Joycelyn Yiu[2,6], Xiaochun Wang[1], Narayan Sahoo[1], Wenhua Cao[1], Xiaodong Zhang[1], Clifford Stephan[3], Radhe Mohan[1], David R. Grosshans[2*], Fada Guan[1*]

[1] Department of Radiation Physics, The University of Texas MD Anderson Cancer Center, Houston, TX 77030, USA

[2] Departments of Radiation and Experimental Radiation Oncology, The University of Texas MD Anderson Cancer Center, Houston, TX 77030, USA

[3] Center for Translational Cancer Research, Texas A&M Health Science Center, Institute of Biosciences and Technology, Houston, TX 77030, USA.

[4] Department of Radiation Oncology, Ruijin Hospital, Shanghai Jiaotong University School of Medicine, Shanghai, 200025, China

[5] Department of Nuclear Science and Engineering, Nanjing University of Aeronautics and Astronautics, Nanjing, 210016, China

[6] Department of BioSciences, Rice University, Houston, TX, 77005, USA

*Corresponding Authors:

Fada Guan, PhD, E-mail: FGuan@mdanderson.org

David R. Grosshans, MD, PhD, E-mail: dgrossha@mdanderson.org

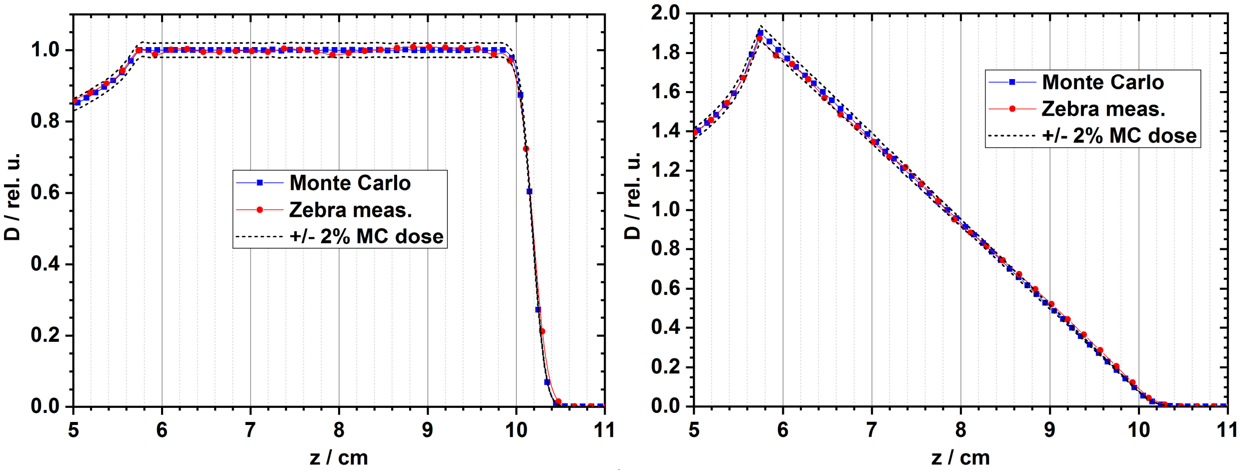

(A) (B)

Figure S1. The validation of depth dose distributions using a Zebra multi-layer ion chamber. (A) The results from a single field flat SOBP. (B) The results from a single filed downslope SOBP. For both setups, the measure dose at any depths in the SOBP is within the +/- 2% lines (black dash) of the Monte Carlo calculated dose.

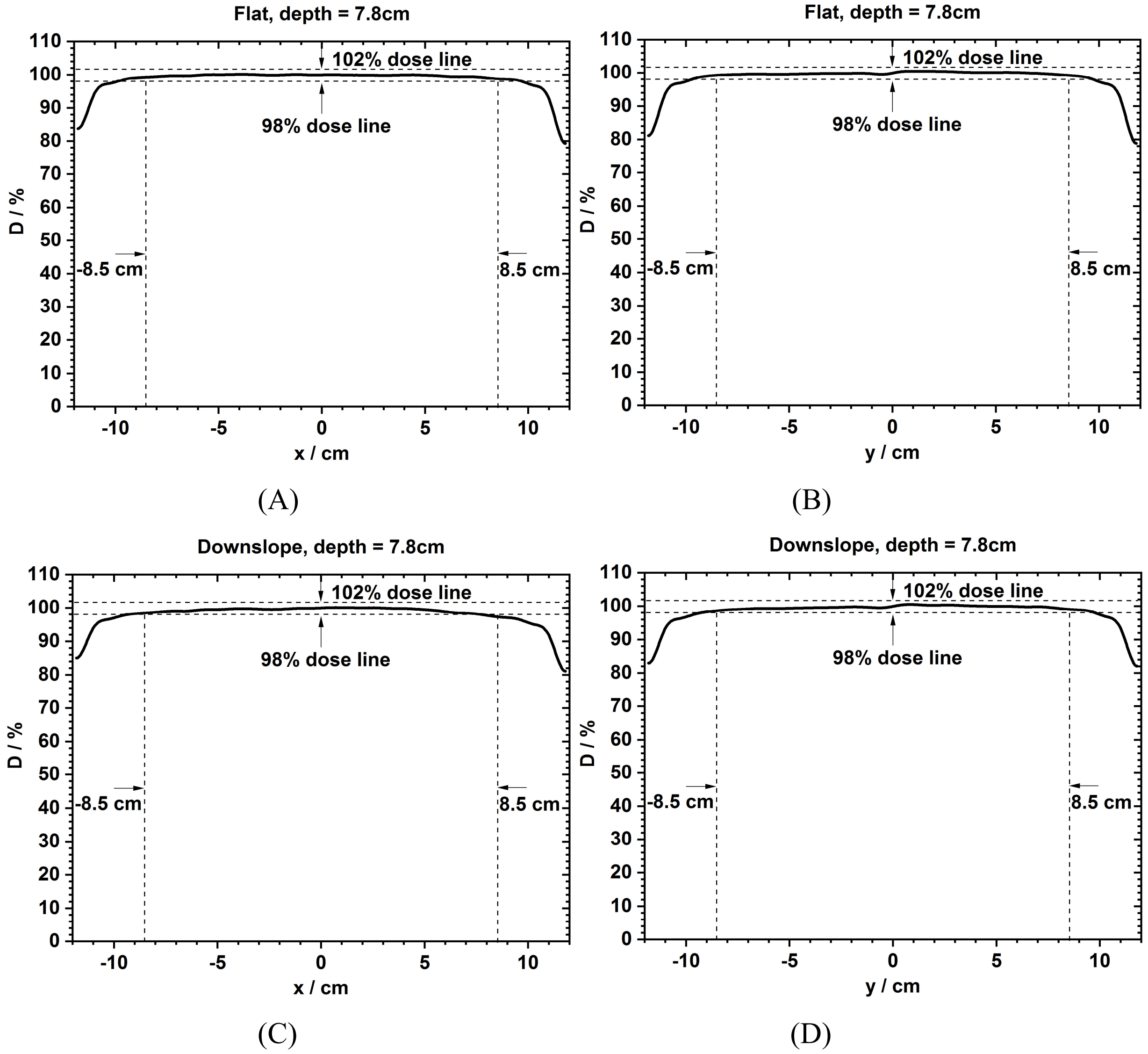

Figure S2. The lateral dose profiles along central x and y axes at the depth of z = 7.8 cm. The 2D dose maps were first measured using a MatriXX ion chamber array. The WET of 7.4 cm solid water blocks were used because the WET of the MatriXX window is 0.4 cm. Then the 1D lateral dose profiles were extracted from the 2D dose data. (A) & (B) The lateral dose profiles along the central x and y axes from a single field flat SOBP. (C) & (D) The lateral dose profiles along the central x and y axes from a single field downslope SOBP. For both setups, the dose uniformity along either x or y axis is within 98% and 102% dose lines. For each setup, the average dose from the central area of 2 cm × 2 cm was used as the normalization factor.

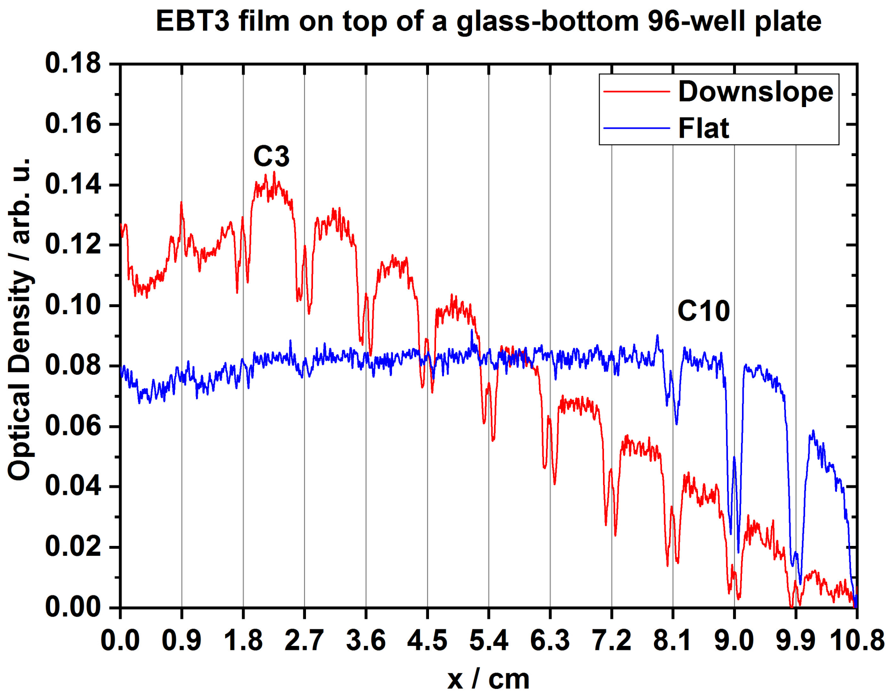

Figure S3. The optical density distributions from EBT3 film measurements within the 12 columns of a 96-well plate with glass bottoms. C3 and C10 stand for the column 3 and 10. The optical density profiles are extracted from the central line crossing the row 4 of the 96-well plate. The resolution of the images is 0.1 mm.

Table S1. The surviving fraction as a function of LET_d for both of scan patterns using two opposed flat fields and two opposed downslope fields. Both of the experimental data and predictions using the McNamara RBE model are listed. For the experimental data, the error is the SEM (n = 3 repeats). For the results using the McNamara model, the error is derived from the error propagation including all the errors of the variables in the McNarama formula and the standard errors of α and β from the survival curve of the reference photons.

**LET_d (keV/μm)**		**Surviving fraction @ 2 Gy**				**Surviving fraction @ 4 Gy**			
		**Experiment**		**McNamara model**		**Experiment**		**McNamara model**	
		**Mean**	**Error**	**Mean**	**Error**	**Mean**	**Error**	**Mean**	**Error**
**Flat**	2.87	0.532	0.024	0.447	0.041	0.118	0.015	0.079	0.019
	2.98	0.532	0.028	0.444	0.041	0.114	0.013	0.078	0.018
	3.23	0.507	0.035	0.436	0.041	0.114	0.010	0.076	0.018
	3.93	0.503	0.038	0.417	0.041	0.113	0.009	0.070	0.017
**Downslope**	4.20	0.457	0.012	0.410	0.041	0.075	0.015	0.067	0.017
	4.49	0.425	0.011	0.403	0.041	0.057	0.010	0.065	0.016
	4.65	0.428	0.024	0.398	0.041	0.059	0.010	0.064	0.016
	4.68	0.423	0.019	0.398	0.041	0.063	0.014	0.064	0.016

Table S2. The number of 53BP1 foci per nucleus (at 2 Gy at 24 hours after irradiation) as a function of LET_d for both flat and downslope fields. The error is SEM. n is the total number of cell nuclei in the images for analysis at each irradiation condition.

	**Flat**				**Downslope**			
**LET_d (keV/μm)**	2.87	2.98	3.23	3.93	4.2	4.49	4.65	4.68
Mean	2.655	2.759	2.761	2.773	2.691	2.807	2.836	2.851
Error	0.018	0.019	0.019	0.019	0.019	0.019	0.020	0.020
n	21710	20805	20933	21649	19816	20673	20138	19994

Table S3. The absolute dose in three selected columns from Monte Carlo simulations and measurements using the Advanced Markus chamber.

	**Flat**			**Downslope**		
**Column #**	Monte Carlo (Gy)	Measurement (Gy)	Difference (%)	Monte Carlo (Gy)	Measurement (Gy)	Difference (%)
**3**	1.00	1.01	1.00%	1.79	1.84	2.79%
**7**	1.00	1.02	2.00%	0.90	0.88	-2.22%
**8**	1.00	1.03	3.00%	0.68	0.70	2.94%

Table S4. The sensitivity analysis of dose variation on the WET change of the irradiation system and 96-well plates.

**Column #**	**Two opposed flat fields**		**Two opposed downslope fields**	
	-1 mm	+1 mm	-1 mm	+1 mm
3&10	-0.07%	-0.09%	4.19%	-4.21%
4&9	-0.05%	-0.01%	4.12%	-4.23%
5&8	0.07%	-0.04%	4.17%	-4.46%
6&7	0.09%	0.09%	4.27%	-4.15%

Table S5. The sensitivity analysis of LET_d variation on the WET change of the irradiation system and 96-well plates.

**Column #**	**Two opposed flat fields**		**Two opposed downslope fields**	
	-1 mm	+1 mm	-1 mm	+1 mm
3&10	-5.85%	8.45%	0.38%	0.96%
4&9	-3.34%	3.33%	-1.76%	0.77%
5&8	-2.50%	1.40%	-2.37%	-0.14%
6&7	-1.99%	2.11%	-1.87%	1.92%

S.1 The proton RBE calculations using the phenomenological McNamara model

The phenomenological McNamara model [1] was used to calculate the proton RBE in this study. The McNamara RBE calculation formula is expressed in Equation (S1).

$$RBE\left[D_p,\left(\frac{\alpha}{\beta}\right)_x,LET_d\right]=\frac{1}{2D_p}\left(\sqrt{\left(\frac{\alpha}{\beta}\right)_x^2+4D_p\left(\frac{\alpha}{\beta}\right)_x\left(p_0+\frac{p_1}{(\alpha/\beta)_x}LET_d\right)+4D_p^2\left(p_2+p_3\sqrt{(\alpha/\beta)_x}LET_d\right)^2}-\left(\frac{\alpha}{\beta}\right)_x\right) \quad \text{(S1)}$$

Here D_p and LET_d are the proton dose and dose-averaged LET. The fitting coefficients can be obtained from the original publication and they are p_0 = 0.99064 (standard error: 0.014125), p_1 = 0.35605 (standard error: 0.015038), p_2 = 1.1012 (standard error: 0.0059972), and p_3 = -0.0038703 (standard error: 0.00091303). The α_x and β_x parameters are the photon SF curve fitting coefficients. In the current study, the results for H460 cells with photon irradiations are provided in section 2.3.

There are three steps in calculating the SF using an RBE model. We first used the McNamara model to calculate the proton RBE based on the dose and LET_d values. Second, we calculated the RBE weighted dose according to the definition that $D_x = RBE \times D_p$. Third, we used the Equation (1) to calculate the SF with D_x, α_x and β_x as input parameters. The calculated cell surviving fractions were then compared with the experimental data from clonogenic assays. The details about the calculations can be found in our previous publication [2].

S.2. The clinical potential of treatment plans with an elevated LET_d distribution

As discussed in the **Introduction**, many groups have developed methods in an effort to enhance the biological effects to the target tumors by applying LET_d-based RBE modeling in plan optimizations. However, because RBE models are not experimentally validated, it is difficult to evaluate the clinical outcome from these plans. Because the RBE factor of 1.1 is still being used clinically and will not be changed in the near future, maintaining the same dose level but increasing the LET_d may be an ideal alternative to enhance the biological effects in the target volume. To evaluate the clinical potential of downslope fields, two research plans for a prostate cancer patient were generated using the two opposed flat and downslope dose fields, shown in **Figure S4**[3]. The plan in **Figure S4A** is a typical plan used in the current clinical practice with two opposed flat dose fields. The plan in **Figure S4B** was created using the criterion with the high-priority of enhancing LET_d in the target. The prescribed dose is 78 Gy(RBE) in 39 fractions. The constant RBE of 1.1 is used in the plans. The clinical target volume (CTV) is contoured by the red line and the planning target volume (PTV) is contoured by the gray line. The dose volume histograms (DVHs) to the CTV and organs at risk (OARs) show that for CTV, rectum, and bladder, the DVHs from both plans are comparable, but for the femoral heads, the dose is higher when

the downslope fields are used. Nevertheless, the DVH of the femoral heads still follows the guideline of IMPT plans for prostate cancers. However, the increase of the dose to femoral heads may be mitigated by applying dose constraints in optimization or adopting more fields than two. The plans shown here are only used for the purpose of research. Ultimately, physicians will need to determine the tradeoff between possible dose degradation and LET_d enhancement in clinical situations. To further evaluate the clinical outcome of using downslope fields, clinical trials are needed.

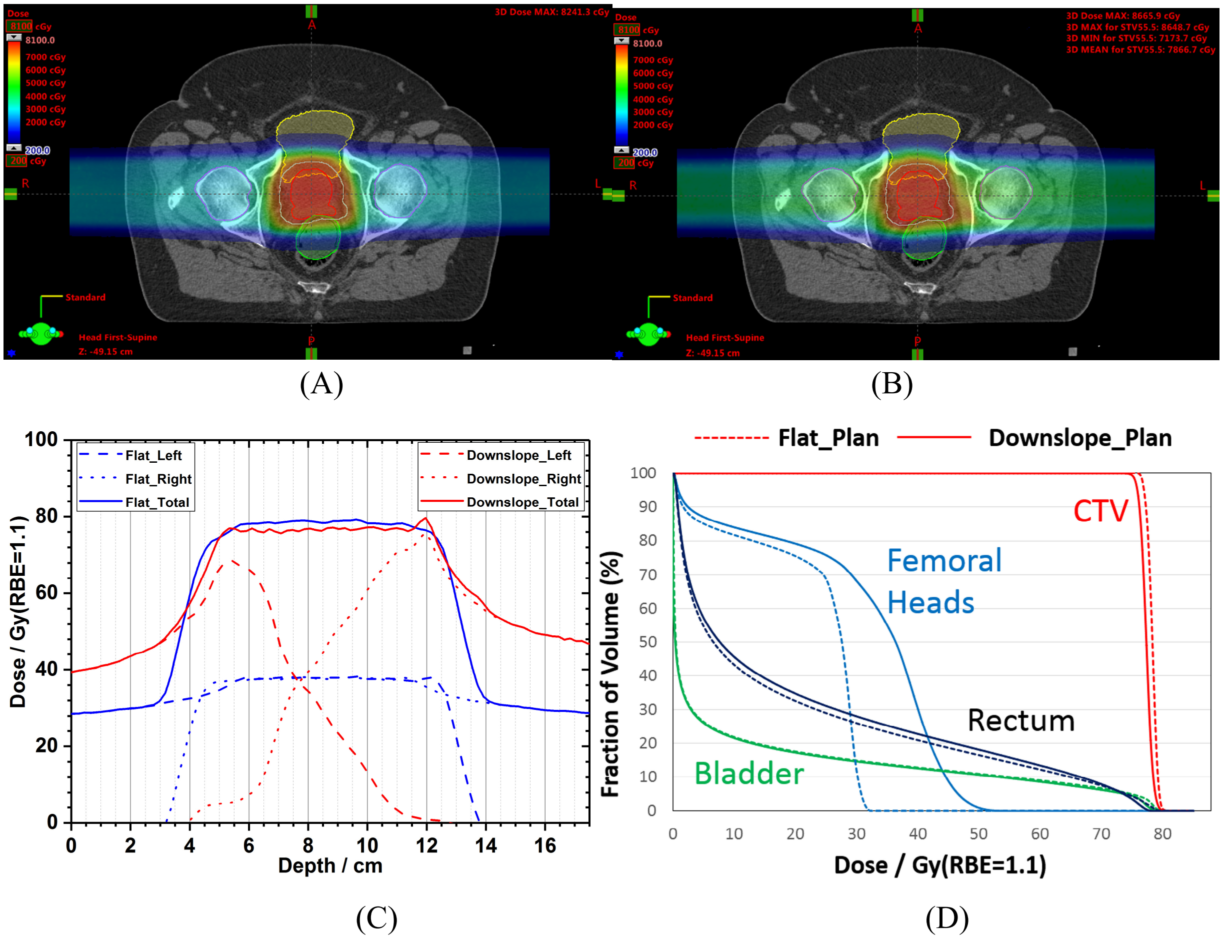

Figure S4. The research treatment plans for a prostate cancer patient. The CTV is contoured by the red line and the PTV is contoured by the gray line. The rectum is contoured by the green line. The bladder is contoured by the yellow line. The femoral heads are contoured by the blue line. The prescribed dose is 78 Gy(RBE) in 39 fractions. The constant RBE of 1.1 is used in the plans. (A) The plan with two opposed flat dose fields. (B) The plan with two opposed downslope dose fields. The plans clearly show that the dose to the femoral heads is higher when two opposed downslope dose fields are used. (C) The dose profiles crossing the middle line of the CTV. (D) The DVHs of the CTV and OARs. The dose to the femoral heads is higher using downslope fields.

References

1 McNamara, A. L., Schuemann, J. & Paganetti, H. A phenomenological relative biological effectiveness (RBE) model for proton therapy based on all published in vitro cell survival data. *Phys Med Biol* **60**, 8399-8416, doi:10.1088/0031-9155/60/21/8399 (2015).

2 Guan, F. *et al.* RBE Model-Based Biological Dose Optimization for Proton Radiobiology Studies. *International Journal of Particle Therapy* **5**, 160-171 (2018).

3 Cao, W. *et al.* Linear energy transfer incorporated intensity modulated proton therapy optimization. *Physics in Medicine & Biology* **63**, 015013 (2017).
